# Polarization-sensitive optical coherence tomography–based fully-automated volumetric coronary fibrous cap characterization

**DOI:** 10.1093/ehjimp/qyag076

**Published:** 2026-04-27

**Authors:** Georgia L Jones, Kenichiro Otsuka, Laurens van Zandvoort, Joost Daemen, Brett E Bouma, Martin Villiger

**Affiliations:** Wellman Center for Photomedicine, Massachusetts General Hospital, Boston, MA 02139, USA; Institute for Medical Engineering and Science, Massachusetts Institute of Technology, Cambridge, MA, USA; Department of Cardiovascular Medicine, Osaka Metropolitan University Graduate School of Medicine, Osaka, Japan; Department of Cardiology, Thoraxcenter, Erasmus University Medical Center, Rotterdam, The Netherlands; Department of Cardiology, Thoraxcenter, Erasmus University Medical Center, Rotterdam, The Netherlands; Wellman Center for Photomedicine, Massachusetts General Hospital, Boston, MA 02139, USA; Institute for Medical Engineering and Science, Massachusetts Institute of Technology, Cambridge, MA, USA; Wellman Center for Photomedicine, Massachusetts General Hospital, Boston, MA 02139, USA

**Keywords:** atherosclerosis, fibrous cap thickness, optical coherence tomography, polarimetry, convolutional neural network

## Abstract

**Aims:**

Accurate characterization of fibrous caps in coronary arteries allows more precise estimations of coronary plaque rupture risk. While intravascular imaging techniques offer high-resolution imaging, limitations in contrast and reliance on manual interpretation hinder large-scale, volumetric assessments of cap morphology. In this study, we present CapSeg, a fully automated computational pipeline for *in vivo* fibrous cap segmentation and thickness measurement, leveraging intravascular polarization-sensitive OCT (IV-PS-OCT). By incorporating reflectance, birefringence, and depolarization metrics, CapSeg differentiates fibrous caps from lipid-rich cores, enabling automated extraction of minimum cap thickness and polarization properties across entire vessel segments.

**Methods and results:**

Using a dataset of 200 cross-sectional coronary IV-PS-OCT images, CapSeg’s minimum cap thickness measurements were validated against manual annotations from two expert observers. Automated cap thickness measurements showed strong agreement with manual assessments (mean ± SD: CapSeg 131 ± 80 µm; Observer 1: 137 ± 84 µm; Observer 2: 144 ± 83 µm) demonstrating comparable limits of agreement relative to the inter-observer variability. The pipeline was applied to volumetric IV-PS-OCT data of 38 coronary lesions from patients with acute coronary syndrome (ACS, *n* = 23) or chronic coronary syndrome (*n* = 15). This analysis revealed decreased birefringence (3.7·10^−4^ vs. 4.5·10^−4^) and increased depolarization (9.6·10^−2^ vs. 8.6·10^−2^) in the fibrous caps of patients with acute disease.

**Conclusion:**

Overall, CapSeg enables fast, reproducible, and fully automated fibrous cap evaluation, laying the foundation for large-scale clinical studies and real-time intravascular imaging applications.

## Graphical Abstract

**Graphical Abstract:**
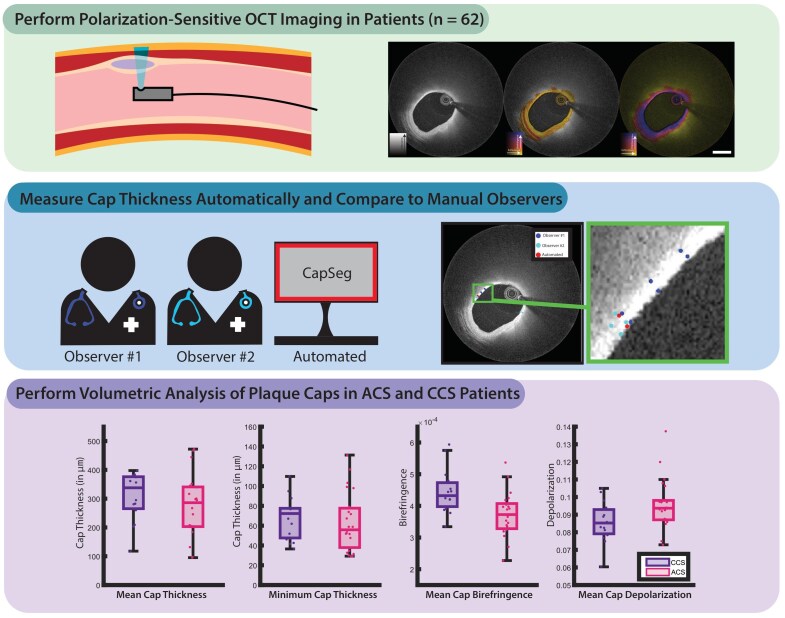

## Introduction

Intravascular optical coherence tomography (IV-OCT)^1–3^ enables detailed investigation of coronary plaque structure^1–5^ and is routinely used to guide percutaneous coronary intervention.^1,5,6^ Polarization-sensitive IV-OCT (IV-PS-OCT) is a modification of conventional IV-OCT, complementing the high-resolution structural information of IV-OCT with polarimetric measurements of tissue properties, including birefringence and depolarization.^7,8^ In birefringent tissues, the refractive index varies with the direction and polarization of light. These tissues are typically fibrillar in nature and include components such as collagen and smooth muscle cells.^9,10^ Depolarization refers to the loss of a uniform polarization state due to light–tissue interactions.^7,8^ This property is typically elevated in tissues that exhibit multiple scattering, such as lipid-rich areas and necrotic core material.^10^ Both birefringence and depolarization provide additional contrast, which allows for more detailed plaque characterization.^11–14^

Fibrous cap composition and thickness are critical markers of plaque vulnerability.^15–19^ In addition, these properties are increasingly clinically important when assessing plaque stability following lipid-lowering therapies, including statins and PCSK9i.^20–23^ However, manual segmentation is labour-intensive, requires training, and introduces variability that limits large-scale analyses and clinical implementation.^24^

Our group previously developed OCTSeg, a convolutional neural network designed to segment major coronary structures and plaque regions in IV-PS-OCT imaging.^25^ While effective for plaque identification, the model did not incorporate segmentation of fibrous caps. Given that IV-PS-OCT provides intrinsic contrast between collagen-rich caps and lipid-rich cores, the modality is particularly well suited for automated and quantitative assessment of fibrous cap morphology.

In this study, we leveraged the enhanced contrast capabilities of IV-PS-OCT for lipid and collagen, in conjunction with our OCTSeg framework, to achieve fully automated segmentation of fibrous caps and precise measurements of minimum cap thickness. Furthermore, we employed this framework for automated volumetric analysis for comprehensive comparison of cap thickness and compositional profiles in target lesions from patients with acute coronary syndrome (ACS) and chronic coronary syndrome (CCS) in a retrospective study.

## Methods

### Study population and consent

IV-PS-OCT pullbacks from 62 patients were acquired during routine PCI in two prior studies at Erasmus University Medical Centre between 2014 and 2018.^13,14,20–23^ The study was approved by the Ethics Committee of the Erasmus University Medical Centre, and all patients gave written informed consent.

For comparison with manual measurements, cross-sections with fibroatheroma and lipid arc >90° were preselected. Caps were visually categorized as thin (<80 µm) or thick. Seventy-two thin and 1227 thick sections were identified, and 200 (all thin plus randomly selected thick) were retained for this comparison.

### PS-OCT system

The IV-PS-OCT system used to collect the clinical data employed commercial intravascular catheters (FastView, Terumo) connected to a custom PS-OCT system, described previously in detail.^11–14,26^ In short, the system used a wavelength-swept light source with a repetition rate of 104 kHz centred at 1300 nm with a scanning range of 110 nm, resulting in a full-width at half maximum of the axial point spread function of 9.4 µm in tissue. To enable polarization sensitivity, the system incorporated polarization diverse detection and an electro-optic modulator in the sample arm, alternating the polarization between states located at a relative angle of 90° on the Poincaré sphere. The catheter provided a lateral resolution of 35 µm, performing 100 rotations per second to acquire frames consisting of 1024 depth-scans, while pulling back at a speed of 20 mm/s.

### Manual fibrous cap thickness measurements

Minimum fibrous cap thickness measurements were manually performed in QCU-CMS (version 4.63, Leiden University Medical Centre, Leiden, The Netherlands) by two cardiologists (K.O., L.v.Z.) according to the consensus standard of OCT interpretation^3^ and blinded to clinical presentation. Only the structural OCT images were used. The 200 cross-sections were each shown three times in randomized order and with randomly rotated orientations to evaluate both intra- and inter-observer variability.

### Automated segmentation network (OCTSeg)

**Figure 1:**
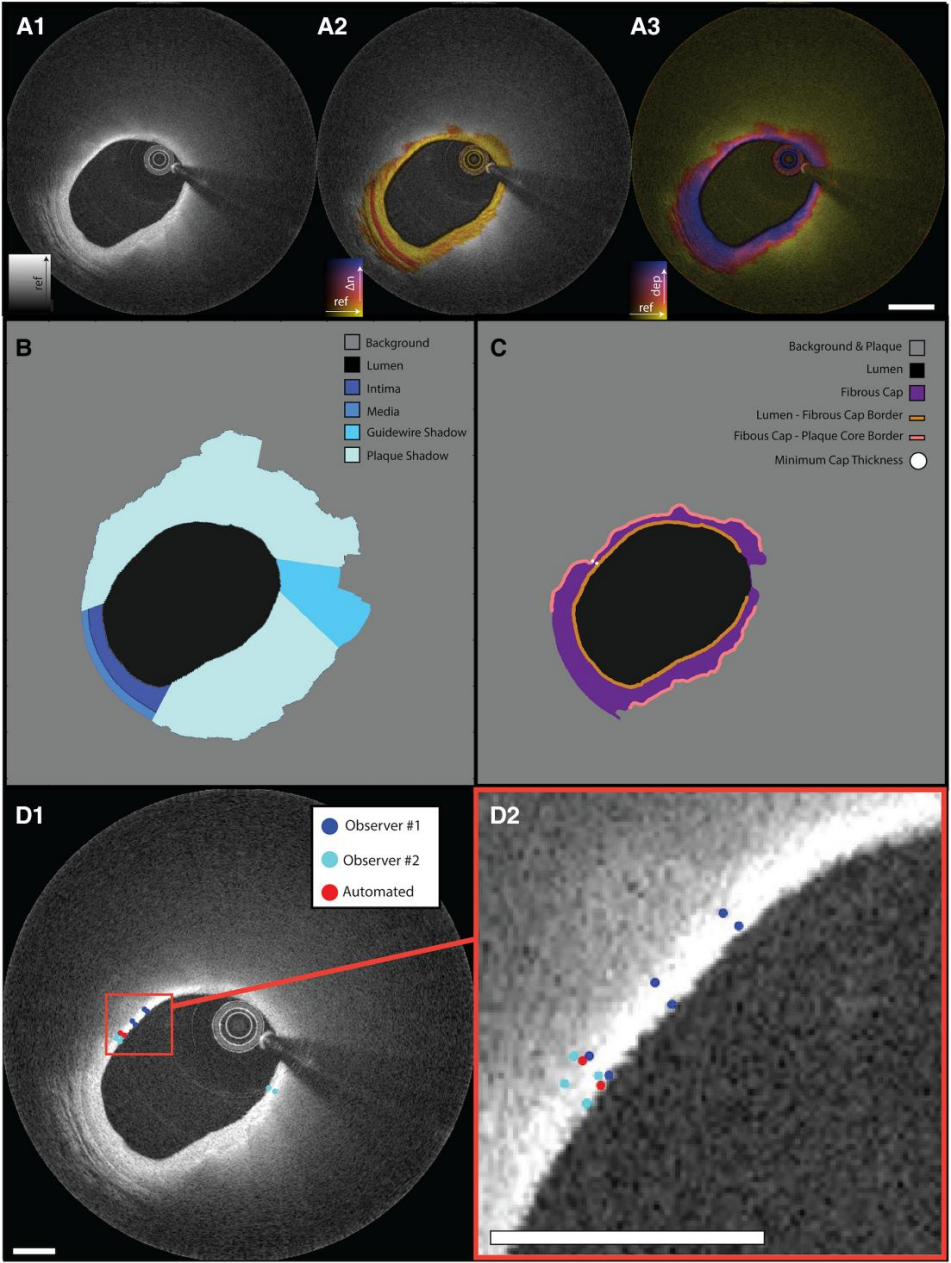
Description of image processing pipeline used to generate fully automated cap thickness measurements. The three primary polarization property images a.1) reflectance, a.2) birefringence, and a.3) depolarization are used as input for the OCTSeg network, which outputs the (*B*) segmentation map. The segmentation map identifies areas corresponding to the background, lumen, intima and media as well as artefacts such as the guidewire shadow and plaque shadow. Combining information from the polarization properties (*A*) and the segmentation map (*B*), a map of the fibrous cap is generated and displayed in c. The inner and outer border of the cap within the plaque regions is denoted in (*C*). The dots in (*C*) represent the identified position of the inner and outer boundary of the fibrous cap corresponding to the minimum fibrous cap thickness. The manual segmentation and automated segmentation are compared in a d.1) full view and d.2) magnified view. Scale bar = 1 mm.

For each cross-section, the corresponding reflectance, birefringence and depolarization images were provided as input into the automated segmentation network, OCTSeg, which is described in detail in our previous work.^25^ OCTSeg generates a segmentation mask with 6 labelled areas, corresponding to the segmented background, lumen, intima, media, guidewire shadow and plaque shadow. Plaque shadow highlights areas where the normal three-layer structure of intima, media and adventitia is not clearly imaged by OCT. The intima and media are only segmented in normal areas where the layers are distinguishable, as shown in *Figure 1*.

### CapSeg: automated fibrous cap thickness assessment

Fully automated minimum cap thickness measurements were achieved through a multi-step computational pipeline, denoted in this work as CapSeg. All computations were performed in Cartesian coordinates. Plaque regions were delineated using OCTSeg’s plaque shadow segmentation, which highlights both lipid-rich and fibrous plaques.^25^ Secondly, the relative angle between the intimal surface and the probe beam was computed. At locations where the tissue surface tangent is within ±5 degrees to the imaging angle, there is reduced backscattering, resulting in low reflectance signal and erroneously high depolarization. These probe angles were removed from the delineated plaque together with the guidewire shadow. After the measurable plaque area had been identified, the IV-PS-OCT metrics were used to isolate the fibrous cap from the lipid core. This was done with a combined reflectance and depolarization threshold


Cap=Depolarization<0.2.orReflectance>35dB

where the reflectance is expressed in decibels (dB) above the noise floor and depolarization is on a scale of 0 to 1 with 1 describing fully depolarized light and 0 describing fully polarized light. The depolarization threshold was determined based on previous histology studies investigating the depolarization and reflectance signature of lipid-rich plaques, while the reflectance threshold was empirically determined to improve cap detection in low-signal areas. After the angular range corresponding to the fibrous cap was identified in each cross-section, an inner and outer boundary point of the fibrous cap was determined for each probe angle within the plaque shadow. The outer boundary point was determined to be the maximum depth of the fibrous cap mask, according to the above threshold, while the inner boundary point was determined to be the lumen-fibrous cap intersection. The corresponding minimum cap thickness was measured as the smallest possible distance between any point on the fibrous cap inner boundary and any point on the fibrous cap outer boundary in Cartesian coordinates.

### Volumetric analysis of fibrous caps

Fully automated fibrous cap evaluation allows the opportunity to achieve volumetric assessments of full plaque regions. Target coronary plaques were identified in a subset of patients from the retrospective dataset, with one lesion identified per patient. Patients were excluded if (1) no unstented, clearly discernible plaque was present or (2) image quality was inadequate due to suboptimal contrast flushing. The final study cohort included 38 target plaques, categorized according to clinical presentation as CCS (*n* = 15) or ACS (*n* = 23). Lesions were determined by identifying the location of the minimum lumen area (MLA) of each imaged coronary artery and selecting a pullback segment comprising the MLA cross-section and extending proximally and distally to cross-section with minimal disease. Each cross-section within the identified area was then fed through the CapSeg pipeline to segment and characterize cap regions. Volumetric assessment of the fibrous cap thickness profile and composition using IV-PS-OCT metrics were then performed and compared between CCS and ACS patients. Polarization properties were evaluated over the entire cap volume, with the mean minimum cap thickness, overall minimum cap thickness, mean birefringence and mean depolarization. Birefringence values were only considered in areas with sufficiently low depolarization (<0.2) to ensure accurate measurements.

### Statistical analysis

Bland–Altman plots were generated to evaluate inter- and intra-observer variability in minimum cap thickness measurements. Inter-observer variability was analysed by comparing measurements between the two manual annotators as well as comparing each annotator to the automated framework. Additionally, intra-observer variability was assessed for both annotators by comparing their repeated measurements. As each cross-section was assessed by each manual observer three independent times, to establish intra-observer variability each measurement was compared to the other two measurements, leading to three comparisons in total. For manual vs. automated comparisons, the automated measurement for each cross-section was individually compared to the corresponding three manual measurements, leading to three comparisons for every cross-section.

Group comparisons in volumetric analysis used two-sample Kolmogorov–Smirnov tests with *P* < 0.05 indicating significance.

## Results

### Qualitative minimum cap thickness results

**Figure 2:**
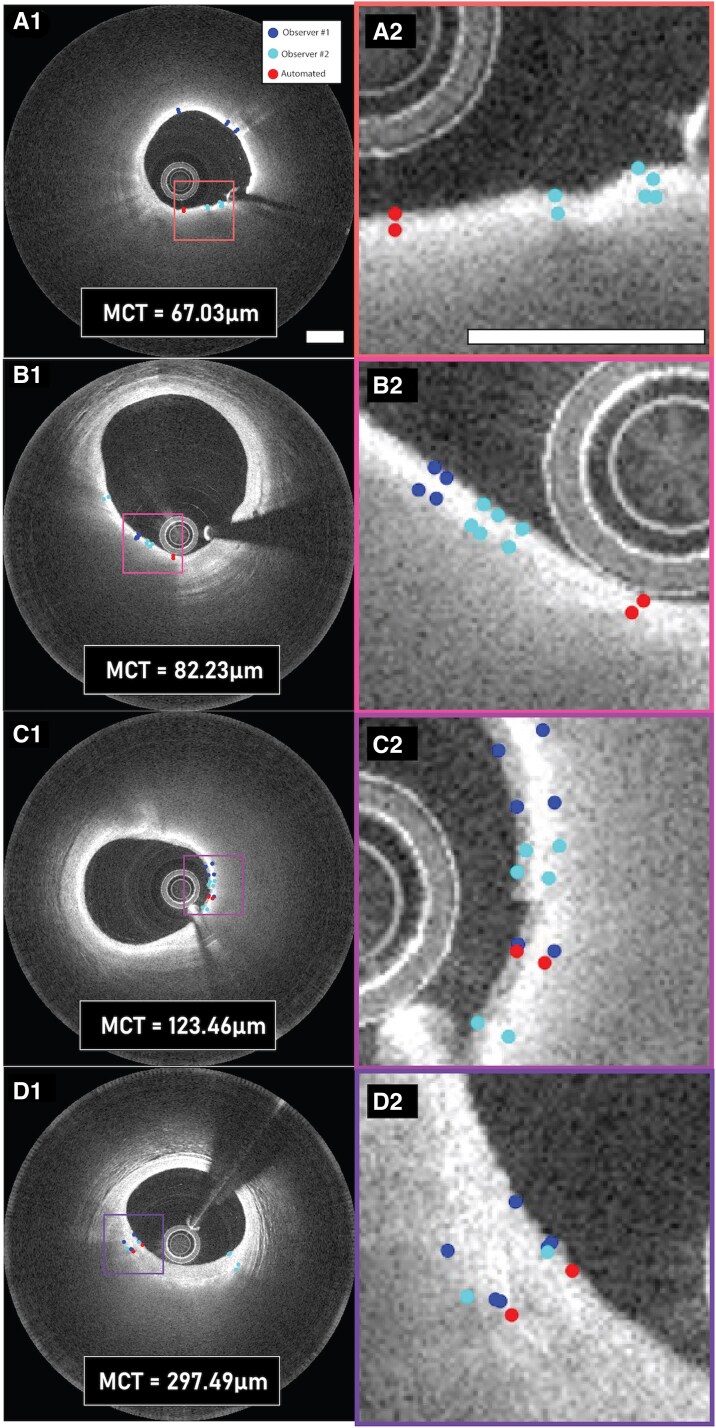
Qualitative results of minimum cap thickness measurements. Start and end points of the minimum cap boundary at the position of the minimum cap thickness are shown for Observer #1, Observer #2 and the automated segmentation framework. Mean manual minimum cap thickness values for each cross-section are displayed at the bottom centre of the leftmost image for each cross-section.

Clinical characteristics are summarized in *Table 1*. A qualitative illustration of CapSeg’s minimum cap thickness output compared with manual annotations is shown in *Figure 2*, which presents qualitative examples of minimum cap thickness (MCT) measurements across four representative OCT cross-sections. The automated measurement of the minimum cap thickness shows close correlation with the manual segmentations shown for a wide range of measured cap thicknesses, from 67.03 µm to 297.49 µm.

**Table 1:** Patient characteristics

	All Patients (*n* = 62)	ACS (*n* = 44)	CCS (*n* = 18)
Age (years) mean ± SD	63.66 ± 10.50	63.34 ± 11.04	64.44 ± 9.27
Male, *n* (%)	43 (69.4%)	26 (59.1%)	17 (94.4%)
Cardiovascular risk factors			
Diabetes, *n* (%)	16 (25.8%)	12 (27.3%)	4 (22.2%)
Hypertension, *n* (%)	37 (59.7%)	30 (68.2%)	7 (38.9%)
Dyslipidaemia, *n* (%)	28 (45.2%)	20 (45.5%)	8 (44.4%)
Smoking, *n* (%)	22 (35.5%)	19 (43.2%)	3 (16.7%)
Medical history			
Prior MI, *n* (%)	16 (25.8%)	13 (29.5%)	3 (16.7%)
Prior PCI, *n* (%)	25 (40.3%)	17 (38.6%)	8 (44.4%)
Prior CABG, *n* (%)	5 (8.1%)	2 (4.5%)	3 (16.7%)
PCI indication			
STEMI, *n* (%)	3 (4.8%)		
NSTEMI, *n* (%)	27 (43.5%)		
Unstable angina, *n* (%)	14 (22.6%)		
Stable angina, *n* (%)	18 (29.0%)		

ACS, acute coronary syndrome; CABG, coronary artery bypass grafting; MI, myocardial infarction; NSTEMI, non-ST elevated myocardial infarction; PCI, percutaneous coronary intervention.

### Minimum cap thickness measurements summary

**Figure 3:**
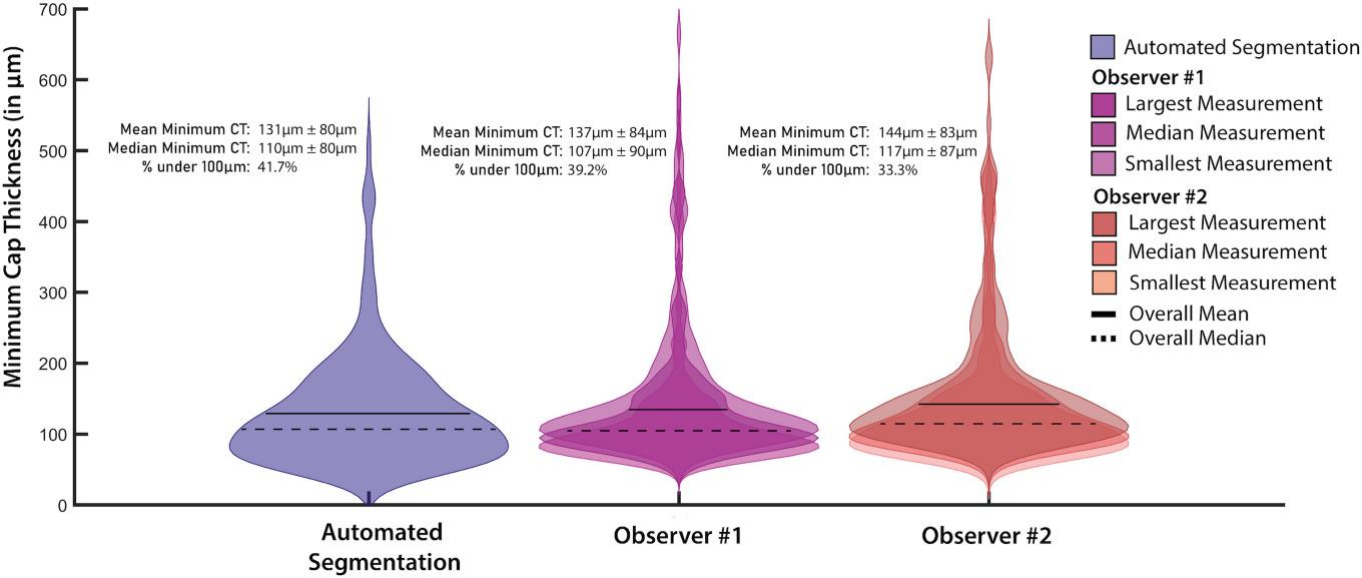
Summary of minimum cap thickness measurements for the manual and automated observers. Violin plots visualize data distributions by displaying the probability density function for each data group. The above violin plots display the minimum cap thickness measurements for the automated observer, Observer #1 and Observer #2. The manual observers estimated the cap thickness three times for each unique cross-section. The minimum, median, and maximum measurements for each unique cross-section are separated into three violin plots within the same axis. The maximum measurement corresponds to the darkest coloured violin plot and the minimum measurement corresponds to the lightest coloured. The overall mean and median of the minimum cap thickness measurements are displayed on the histogram with a solid line and a dashed line black, respectively. The overall mean minimum cap thickness, median minimum cap thickness with standard deviations as well as the percentage of cap thickness measurements under 100 µm are displayed to the left of the histogram.

The results of the minimum cap thickness assessment for each of the manual observers as well as the automated observer are presented in *Figure 3*. Manual measurements yielded a slightly higher overall mean thickness compared with automated methods as well as a slightly higher standard deviation. The automated method identified a higher percentage of thin caps below a 100 µm threshold compared with manual measurements. Percentage of detected caps below 95 µm, 80 µm, and 65 µm are displayed in *Table 2*.

**Table 2:** Thin cap distribution for each observer

% of MCT values	Observer #1	Observer #2	Automated observer
<65 µm	3.8%	8.6%	11.4%
<80 µm	15.2%	29.1%	31.4%
<95 µm	44.8%	61.0%	42.9%

MCT, minimum cap thickness.

### Minimum cap thickness inter-observer and intra-observer variability

**Figure 4:**
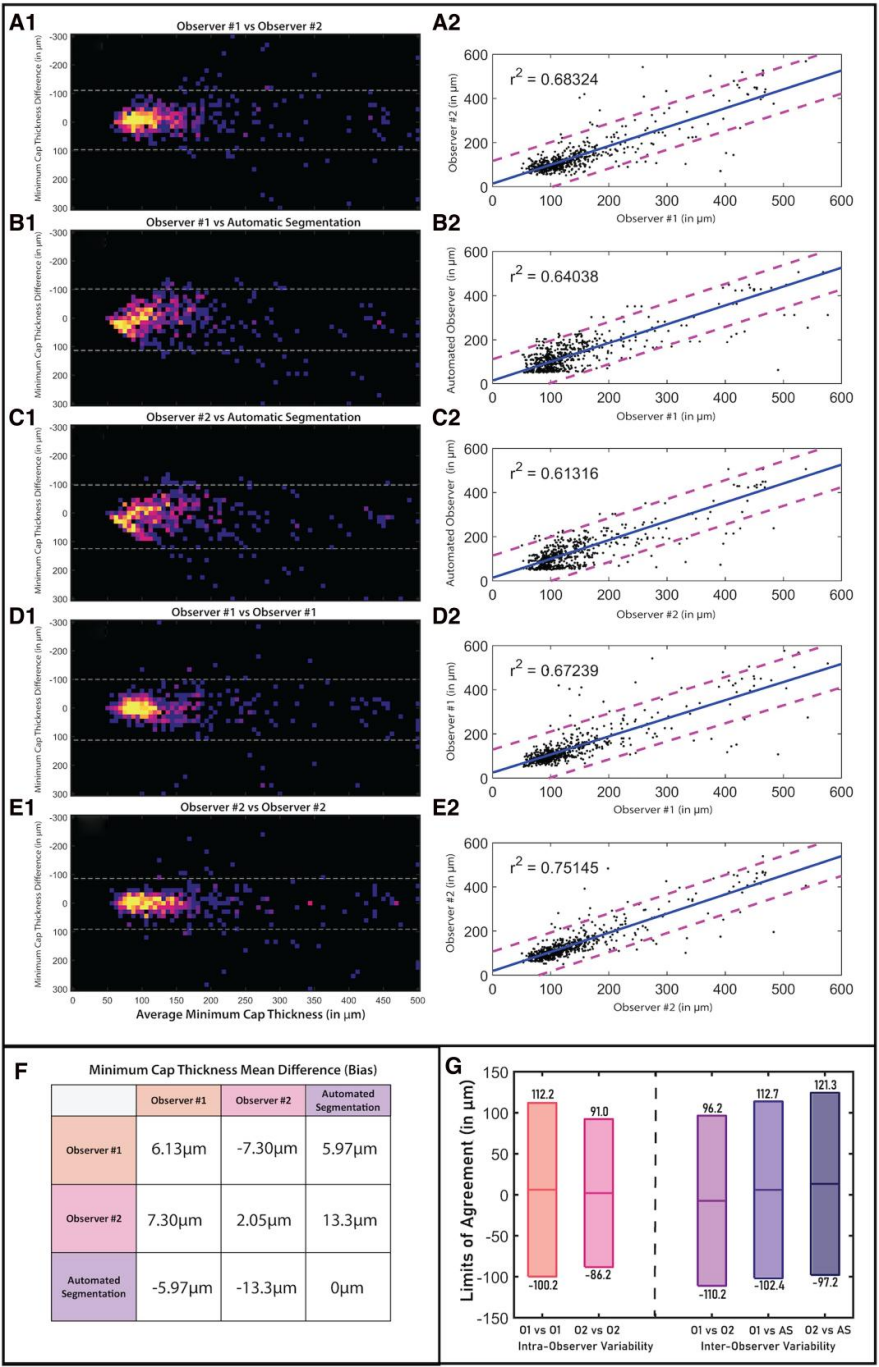
Quantitative results of minimum cap thickness measurements. Bland–Altman plots (1) and scatter plots (2) are shown for all combinations of inter-observer and intra-observer variability, including (*A*) Observer #1 vs. Observer #2, (*B*) Observer #1 vs. Automated, (*C*) Observer #2 vs. Automated, (*D*) Observer #1 vs. Observer #1 and (*E*) Observer #2 vs. Observer #2. Bias values derived from Bland–Altman plots are presented in (*F*), while the limit of agreement ranges are shown in (*G*), with the upper/lower bound of the 95% LOA displayed above/below the range, respectively.

Between the two manual annotators, the mean difference was 7.30 µm, with 95% LoA ranging from −110.0 µm to 96.2 µm, indicating low variability and occasional outliers. Between manual annotators and the automated pipeline, the 95% LoA ranged from −102.4 µm to 112.7 µm and −97.2 µm to 121.3 µm, respectively. This shows a very limited increase in the range of the limit of agreement between the automated pipeline vs. the two manual observers, from 206.1 µm to 215.0 µm and 218.5 µm for the first and second observer, respectively. As shown in *Figure 4*, the minimum cap thickness measurements of Observer #2 were the largest and displayed a positive bias of 6.86 µm compared to Observer #1 and a positive bias of 12.01 µm against the automated observer. The automated framework, on the other hand, had the smallest overall estimations for the minimum cap thickness, with negative biases of −5.15 µm compared to Observer #1 and −12.01 µm vs. Observer #2.

Intra-observer Bland–Altman analysis showed mildly reduced limits of agreement compared to inter-observer variation. The 95% LoA ranged between −100.2 µm and 112.2 µm for the first observer and −86.2 µm and 91.0 µm for the second observer. The biases between these measurements were 6.01 µm and 2.41 µm for the first and second observer, respectively.

### Volumetric analysis of coronary fibrous cap properties

Segmented lesion characteristics, as determined by conventional OFDI, are described in *Table 3*. There were no statistically significant differences reported in the imaged vessel frequency, lesion length and MLA for the ACS vs. the CCS patients.

**Table 3:** Lesion characteristics of volumetric plaque analysis

	All (*n* = 38)	ACS (*n* = 23)	CCS (*n* = 15)	*P*-value
Lesion length (mm)	22.4 ± 7.0	21.9 ± 6.4	20.5 ± 8.0	0.788
Minimum lumen area (mm^2^)	1.8 ± 1.0	1.6 ± 0.8	2.3 ± 1.2	0.097
Imaged vessel				0.976
RCA *n* (%)	18 (47.4%)	11 (47.8%)	7 (46.7%)	
LCA *n* (%)	12 (31.6%)	7 (30.4%)	5 (33.3%)	
LCX *n* (%)	8 (21.0%)	5 (21.8%)	3 (20%)	

ACS, acute coronary syndrome; CCS, chronic coronary syndrome.

**Figure 5:**
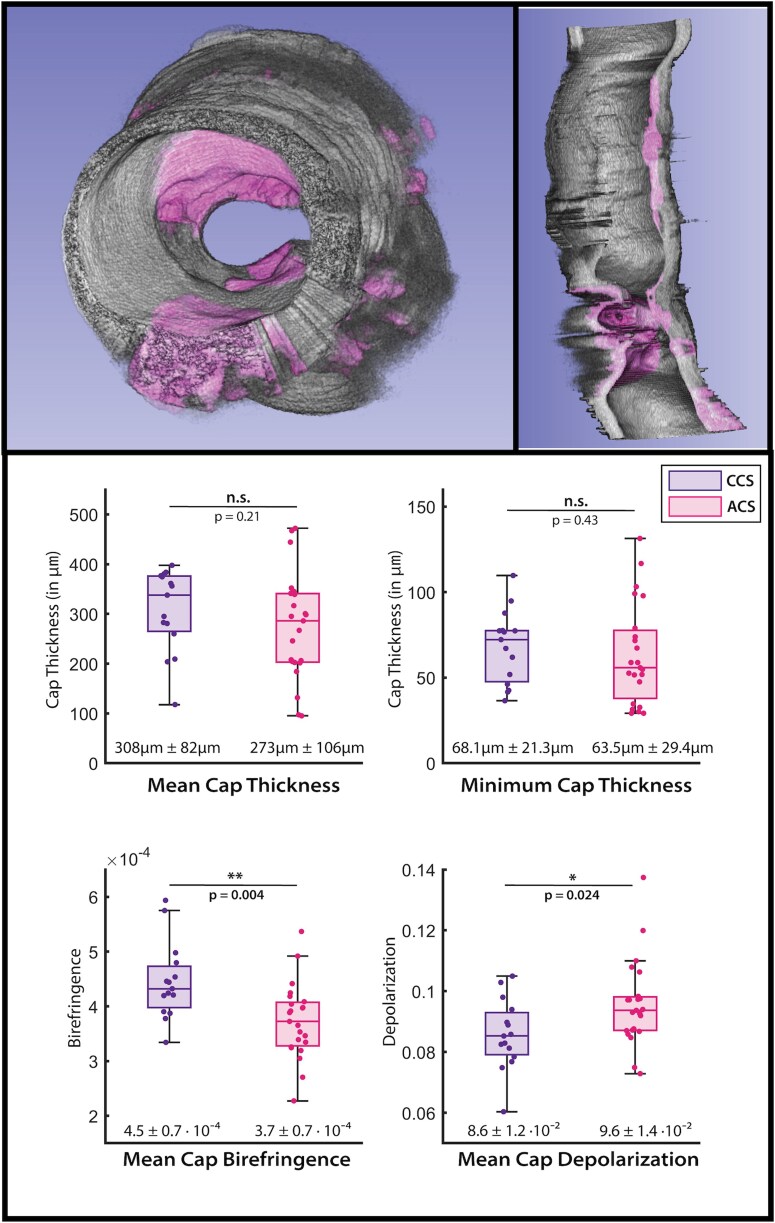
Results of the volumetric cap thickness study comparing cap properties between patients with acute coronary syndrome (ACS) and chronic coronary syndrome (CCS). Renderings of the segmented fibrous caps are shown in (*A*) and (*B*). Volumetric fibrous cap properties are compared in using box plots with overlaid scatterplots (*C*), comparing ACS (right, *n* = 23) and CCS (left, *n* = 15) patients. Mean minimum cap thickness (c.1), absolute minimum cap thickness (c.2), mean cap birefringence (c.3), and mean cap depolarization (c.4) were compared. Statistically significance and corresponding *P*-values are displayed above the box plots. Mean values and standard deviation are presented below the box plots.

The volumetric minimum cap thickness, the mean of the cross-sectional minimum cap thickness, the mean cap birefringence and the mean cap depolarization were found for all 38 target lesions and compared between patients with an indication of acute coronary syndrome (ACS, *n* = 23) and patients with chronic coronary syndrome (CCS, *n* = 15). The results from the volumetric analysis are summarized in *Figure 5*, which displays the mean minimum cap thickness and minimum cap thickness of the volumetric fibrous cap, as well as the mean birefringence and mean depolarization of the fibrous cap over the plaque region.

Statistically significant (*P* < 0.05) differences were found between the birefringence in fibrous caps between ACS and CCS patients. CCS fibrous caps were found to have higher birefringence, with an overall mean of 4.5 · 10^−4^compared with ACS fibrous caps with an overall mean of 3.7 · 10^−4^. Additionally, statistically significant (*P* < 0.05) differences were found between the depolarization within fibrous caps. ACS fibrous caps had slightly higher depolarization values, with an overall mean of 0.096, compared to CCS fibrous caps with an overall mean of 0.086. Minimum and mean cap thickness values did not show statistically significant differences between ACS and CCS patients.

## Discussion

Studies investigating the impact of fibrous cap thickness on plaque stability using IV-OCT have largely focused on single cross-sectional analyses.^15–20,22,24,27^ Generally, these studies involve identifying the location of the minimum lumen diameter,^18^ and measuring the fibrous cap thickness within this section, or in defined cross-sections through the vessel segment containing the plaque.^17^ This process is time-consuming, taking on the order of a few minutes per minimum cap thickness value, labour-intensive, and may be error-prone due to the evaluation of usually a single cross-section.^24^ Additionally, the morphology and thickness of the fibrous cap can vary significantly along the length of the plaque, making single-section assessments potentially insufficient for capturing the full complexity of plaque stability.^28^ To this end, there have been significant efforts towards the development of automated and semiautomated tools to reduce the time burden on researchers and physicians and decrease inter- and intra-observer variability. These studies have incorporated a wide variety of segmentation methods,^24,29–33^ including a recent demonstration of fully automated fibrous cap thickness based on conventional OCT images.^33^

CapSeg has the potential to improve the utility of cap thickness measurements in several ways. Firstly, CapSeg, by capitalizing on the additional information available with PS-OCT, can be performed in a fully automated fashion, removing the need for user intervention and bypassing the potential of user variability. Compared with manual segmentation, CapSeg determines a minimum cap thickness value in a few seconds per cross-section and requires no user intervention or manual editing, allowing for user-independent processing. Additionally, CapSeg allows for the potential of volumetric analysis to better elucidate plaque fibrous cap properties and areas of potential vulnerabilities. The polarization properties extracted from IV-PS-OCT additionally allow for compositional assessments of fibrous caps, potentially revealing markers of instability beyond simple cap thickness.

Previous studies have shown that patients with CCS, or stable angina, display statistically significant higher birefringence and lower depolarization when analysing cross-sectional images of coronary plaques.^13,14^ In this study, we refined the analysis by restricting the measurements to the segmented fibrous cap rather than the entire plaque and furthermore by performing volumetric analysis rather than studying only individual cross-sectional images. This analysis revealed that the mean volumetric cap birefringence and depolarization show statistically significant differences between ACS and CCS patients, in agreement with the previously reported trend. This is expected, as the primary source of birefringence in the fibrous cap is collagen. Collagen provides essential mechanical stability to the fibrous cap, helping to prevent rupture. In ACS patients, macrophages exhibit collagenolytic activity, degrading collagen within the cap and weakening its structural integrity. This loss of collagen renders plaques more prone to rupture, independent of cap thickness. In contrast, CCS patients tend to have plaques with preserved collagen content, contributing to higher measured birefringence.^13,14^ Indeed, no statistically significant difference was found between the mean and minimum cap thickness, further highlighting the complementary contrast achieved with polarimetric signatures relative to structural features alone. The observed differences in the fibrous cap birefringence and depolarization between ACS and CCS lesions, despite the absence of significant differences in cap thickness, suggest that compositional features captured by polarimetric contrast may provide complementary and potentially more sensitive indicators of plaque vulnerability. Future work is required to assess these features’ potential as independent biomarkers of fibrous cap stability.

A major limitation of this study is the small number of patients included. No ruptured plaques were among the lesions of the included patients, potentially leading to a bias in ACS features by underestimating vulnerability. Furthermore, OCTSeg, and as a result CapSeg, requires polarization-sensitive metrics in order to provide accurate results. However, IV-PS-OCT has not achieved widespread clinical adoption and primarily remains a research modality. Only a limited number of patients have been imaged to date with IV-PS-OCT, which limits the total possible patient pool.

Another major limitation is the absence of true ‘thin’ caps, which are below 65 µm. While CapSeg demonstrates strong overall agreement with presented expert manual measurements, the performance for identifying clinically critical very thin caps below these established vulnerability thresholds requires further validation in appropriately enriched patient cohorts with adequate representation of thin cap pathology. The analysis presented in this work indicates that the automated observer exhibits a bias towards thinner cap segmentations; however, it remains unclear whether this reflects increased sensitivity or an artefact of the segmentation pipeline. As such, future thin cap validation should ideally incorporate histopathological ground truth to confirm that automated thickness measurements accurately reflect true cap morphology across the full spectrum of cap dimensions. Future studies with larger sample sizes and direct histological correlation are planned and will be essential to establish the reliability of CapSeg for identifying the most vulnerable cap phenotypes.

Because custom IV-PS-OCT systems are scarce, translation of OCTSeg and CapSeg may rely on recent advances in signal processing techniques, which have overcome the hardware modifications conventionally required to incorporate polarization sensitivity in some conventional clinical systems.^34^ This development may allow for IV-PS-OCT to be more accessible to clinical professionals and potentially for increasing the available patient pool in future studies. As clinical adoption of IV-PS-OCT becomes simplified, there are more opportunities to capitalize on the additional metrics available with this technology.

In this study, we demonstrate the feasibility and efficacy of a fully automated framework, CapSeg, for fibrous cap segmentation and thickness measurement using polarization-sensitive optical coherence tomography (IV-PS-OCT). By integrating reflectance, birefringence, and depolarization metrics into our segmentation pipeline and leveraging our OCTSeg convolutional neural network, we achieve precise and reproducible fibrous cap delineation and minimum cap thickness measurements. The automated measurements show strong agreement with expert manual annotations while significantly reducing the time and labour associated with traditional methods. Future validation studies incorporating histopathological evaluation of cap thickness would add value by confirming the structural basis of CapSeg. The ability to perform volumetric assessments enables a more comprehensive evaluation of plaque morphology, capturing variations in cap thickness and composition that may serve as critical indicators of plaque vulnerability. Comparative analysis of chronic and acute coronary syndrome patients reveals differences in cap characteristics that may inform future risk stratification and therapeutic strategies. Future investigations will aim to evaluate whether IV-PS-OCT parameters provide independent prognostic value in assessing plaque vulnerability and predicting clinical events. Overall, CapSeg offers a scalable and objective tool to advance the study of coronary plaque stability, enabling broader clinical and research applications through high-throughput, operator-independent assessment of fibrous cap integrity.

## Lead author biography


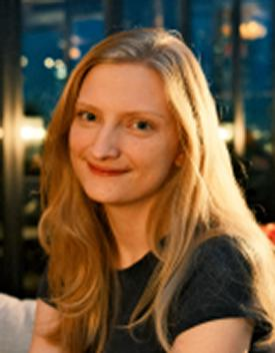
Georgia L. Jones is a Ph.D. candidate in the Medical Engineering and Medical Physics program in the Institute for Medical Engineering and Science at Massachusetts Institute of Technology, USA. She completed her B.Sc. in Honours Physics at McMaster University in Hamilton, Canada. Her research at the Wellman Centre for Photomedicine focuses on advancing polarization-sensitive optical coherence tomography for cardiovascular imaging, including algorithm development and catheter-based system design.

## Data Availability

The datasets generated and analysed during the current study, along with all analysis and processing scripts, are available from the corresponding author upon reasonable request.
